# An Open-Label Pilot Study on Macumax Supplementation for Dry-Type Age-Related Macular Degeneration

**DOI:** 10.1089/jmf.2020.0097

**Published:** 2021-05-17

**Authors:** Muhammed Majeed, Shaheen Majeed, Kalyanam Nagabhushanam

**Affiliations:** ^1^ Sami Labs Limited, Peenya Industrial Area, Bangalore, India.; ^2^ Sabinsa Corporation, Payson, Arizona, USA.; ^3^ Sabinsa Corporation, East Windsor, New Jersey, USA.

**Keywords:** bilberry, lutein/zeaxanthin, piperine, saffron, zinc monomethionine

## Abstract

Age-related macular degeneration (AMD) is one of the most widespread degenerative disorders in elderly people. A 90-day, open-label clinical study was conducted in 40 patients, aged 50 years or older, with early-stage dry-type AMD to evaluate the safety and efficacy of Macumax^®^, a novel mixture of a phyto-mineral nutritional supplement containing ZeaLutein^®^ (consisting of lutein, zeaxanthin, and piperine), extracts of bilberry, saffron, and zinc monomethionine. Subjects received one capsule of the supplement twice daily for 90 days. The treatment measures included physical examination, vital signs, and assessment of subjective and objective symptoms at baseline and after treatment. For efficacy assessment, baseline values were compared with the values after treatment at 30-day intervals, on days 30, 60, and 90. The safety of the treatment was assessed during all the visits. Overall, the patients showed improvement in the subjective symptoms, such as vision scores after treatment compared with baseline. The changes in diminished and distorted vision scores were found to be significant from day 60 (*P* < .05). In the case of objective symptoms, only 40% of the subjects (*P* < .05) had abnormal Amsler's grid aberration scores on day 90 compared with 77.5% of subjects at the beginning of the study. No adverse events were observed during the study. This pilot study provides evidence that Macumax^®^ supplementation is safe and maintained eye health without further progression of the disease in patients with early-stage dry-type AMD. Clinical Trial Registration number: CTRI/2016/02/006676

## INTRODUCTION

Age-related macular degeneration (AMD) is the most widespread degenerative disorder of the central area of the retina (the macula) among people older than 50 years of age. It is a multifactorial disease that causes progressive degeneration of photoreceptors, retinal pigment epithelial (RPE) membrane, and choriocapillaris.^1^ AMD causes blurred central vision due to the thinning of the macula, resulting in permanent vision loss. Globally, people with AMD are anticipated to reach 196 million by 2020 and expected to grow to 288 million by 2040.^2^ Therefore, early detection and preventive measures may delay vision loss due to AMD.

Based on the clinical and pathological features, AMD has been classified into two distinct types, atrophic (dry-type) and exudative (wet-type).^1,3^ Dry-type AMD is described as a gradual loss of the RPE and a further decline in photoreceptors and/or choriocapillaris that leads to loss of vision observed clinically as central and paracentral scotomas.^1,4^ Systemic risk factors include hypertension, smoking, and positive family history.^5^ Obese and lean individuals appear to be at a higher risk for dry-type AMD compared with individuals with normal body mass index (BMI).^6^

Nutritional supplements have become the first line of defense in battling dry-type AMD. Numerous studies have suggested the importance of dietary antioxidants, vitamins, minerals, and micronutrient supplements that retard the progression of AMD.^7^ The first Age-Related Eye Disease Study (AREDS) evaluated the nutritional supplementation that contained antioxidant vitamin C, E, *β*-carotene, zinc, and copper in 55–80 year-old subjects with AMD. The results of the study showed that AREDS supplements were useful in reducing the risk of disease progression in 25% of patients with intermediate or advanced AMD.^8^ A subsequent AREDS2 study conducted using lutein and zeaxanthin, as well as docosahexaenoic acid+eicosapentaenoic acid revealed that the AREDS2 formulation did not reduce the disease progression in patients with advanced AMD.^9^

Dietary xanthophyll carotenoids lutein and zeaxanthin are specifically concentrated in the human macula. Recently, the Eye Disease Case-Control (EDCC) study group showed a direct relationship between the exudative form of AMD and a decreased plasma level of lutein and zeaxanthin.^10^ In another Lutein Antioxidant Supplementation Trial (LAST), AMD patients received a capsule containing either 10 mg of lutein alone or a combination of 10 mg lutein with antioxidants, vitamins, and minerals for 12 months. This study confirmed an improvement in visual function with the supplementation of lutein alone or lutein in combination with other nutrients.^11^

Currently, there is no effective or approved treatment for dry-type AMD. Therefore, there is a crying need for considering suitable nutrients for developing an ideal ocular nutritional supplement, especially for treating patients suffering from early-stage dry-type AMD. Earlier clinical studies have shown that short-term nutritional supplementation is likely to prevent the progression of the disease in patients with early, intermediate, or advanced AMD.^12,13^ This pilot study was aimed at evaluating the efficacy and safety of Macumax^®^, a novel mixture of phyto-mineral nutritional supplements, in patients with early-stage dry-type AMD over a period of 3 months.

## MATERIALS AND METHODS

### Study supplement

The study supplement Macumax^®^ capsules (batch no. ZES00214) were formulated by Sami Labs Ltd. (Bangalore, India) with ZeaLutein^®^ (consisting of zeaxanthin, lutein, and piperine), extracts of bilberry, saffron, and zinc monomethionine, as shown in Table 1. ZeaLutein^®^ granule is a proprietary combination of 1% zeaxanthin, 5% lutein, and 2% piperine, blended in a ratio optimized to provide benefits in eye health. The three natural ingredients are from *Lycium barbarum*, *Tagetes erecta,* and *Piper nigrum*, respectively. The ratio of zeaxanthin to lutein 1:5 in ZeaLutein^®^ is the same as that observed in human plasma. Each manufacturing/packaging process was performed and documented in conformity with Good Manufacturing Practice.

**Table 1.** Formulation of Study Supplement Capsules

No.	Ingredients	Composition
1	ZeaLutein^®^ (lutein: 5 mg+zeaxanthin: 1 mg+piperine: 2 mg)	100 mg
2	Bilberry extract	20 mg
3	Saffron extract	5 mg
4	Zinc monomethionine	7.5 mg

### Study design

**FIG. 1.**
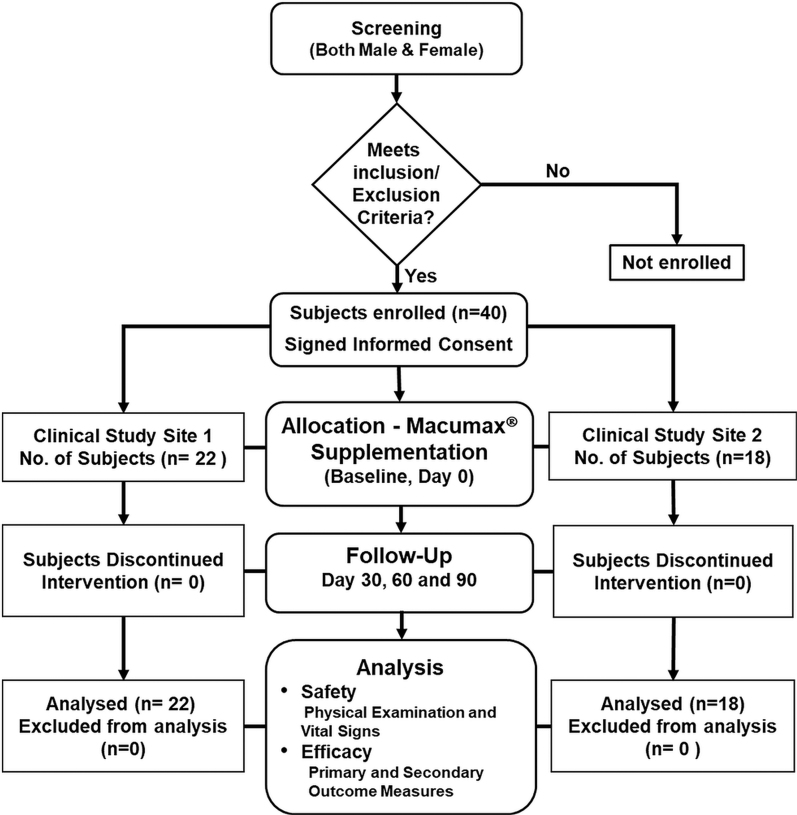
Study design flow diagram of Macumax^®^ supplementation in patients with dry AMD. Of the 40 subjects who completed the study, 22 were screened at Site 1 and 18 were screened at Site 2. No one dropped out. All 40 subjects were included for the safety and efficacy analyses. AMD, age-related macular degeneration.

A total number of 40 subjects with early-stage dry-type AMD were enrolled and the trial was carried out in two independent study sites, Dr. Baba Sahib Ambedkar Medical College & Hospital, Bangalore (Site 1) and Vijaya Super Specialty Hospital, Andhra Pradesh (Site 2) in India from March 11, 2016 to July 11, 2016. Both male and female subjects, more than 50 years of age, who fulfilled the inclusion criteria, were enrolled (Supplementary Table S1). This open-label clinical trial was designed in accordance with the Consolidated Standards of Reporting Trials (CONSORT) guidelines, as shown in the flow diagram (Fig. 1).

### Ethical approval and consents

The study was performed in accordance with the good clinical practice as required by the International Conference on Harmonization, local regulatory requirement, and standard operating procedures for clinical investigation and in compliance with the Declaration of Helsinki. The trial was conducted as per the protocol approved by the Institutional Ethics Committee without any deviation and was registered as a clinical trial (CTRI/2016/02/006676) at a public Clinical Trial Registry in India (www.ctri.nic.in).^14^ Written informed consent was obtained from all the participants of this trial.

### Treatment and efficacy endpoint measurements

All enrolled patients at the two intervention sites were instructed to self-administer one capsule containing the study supplement twice daily for 90 days. Basic demographics such as height, weight, and BMI were determined at screening and after treatment at 30, 60, and 90 days. For efficacy evaluation, subjective symptoms/primary endpoint parameters such as difficulty in day vision, diminished vision, distorted vision, perception of black spots, dark adaptation, perception of flashes of light (xanthopsia graded as +ve/−ve), and perception of objects as yellowish (graded as +ve/−ve) were assessed. The following objective symptoms/secondary endpoint parameters, namely visual acuity to determine the improvement in distant vision and pinhole vision, improvement in the fundus changes, optical coherence tomography (OCT) changes, Amsler's grid aberration changes, and improvement in the visual field by using Humphrey visual field examination, were evaluated. All medications taken by the subjects from the start to the end of the trial were documented to determine the possible interference with the outcome of the study.

### Safety assessment

For safety parameters, physical examination and vital signs were measured at screening and during the treatment period. Any adverse events (AEs) reported by the subjects were also captured on their respective Case Report Forms. All the enrolled subjects completed the study.

### Statistical methods

The data are presented as mean ± standard deviation (SD). Paired *t*-tests were performed to evaluate all the data to arrive at statistical significance on the progressive improvement in subjective and objective symptoms in patients who received the study medication. Results with a *P* value of *<*.05 are considered statistically significant. The Statistical Analysis Software (SAS) 9.1.3 version of the statistical software package (Cary, NC, USA) was used for the analysis of data.

## RESULTS

### Baseline and demographic characteristics

The baseline demographic characteristics are given in Table 2, and the subjects selected for the study were within the normal weight range as per the Center for Disease Control guidelines.^15^

**Table 2.** Patients' Demographic Characteristics Selected for the Trial

Parameter	Statistics	Study supplement
		(*n* = 40)
Age (years)	Mean ± SD	58.97 ± 7.5
	Median	58
	Min; Max	50; 89
Gender, *n* (%)	Male	17 (42.5)
	Female	23 (57.5)
Bodyweight (kg)	Mean ± SD	61 ± 10.7
	Median	60
	Min; Max	38;81
Height (cm)	Mean ± SD	157.8 ± 10.4
	Median	158.5
	Min; Max	137; 180
BMI (kg/m^2^)	Mean ± SD	24.65 ± 3.6
	Median	24.74
	Min; Max	16.23; 36.22

Data presented as mean ± SD.

Max, maximum; Min, minimum.

BMI, body mass index; SD, standard deviation.

### Primary efficacy outcomes

**FIG. 2.**
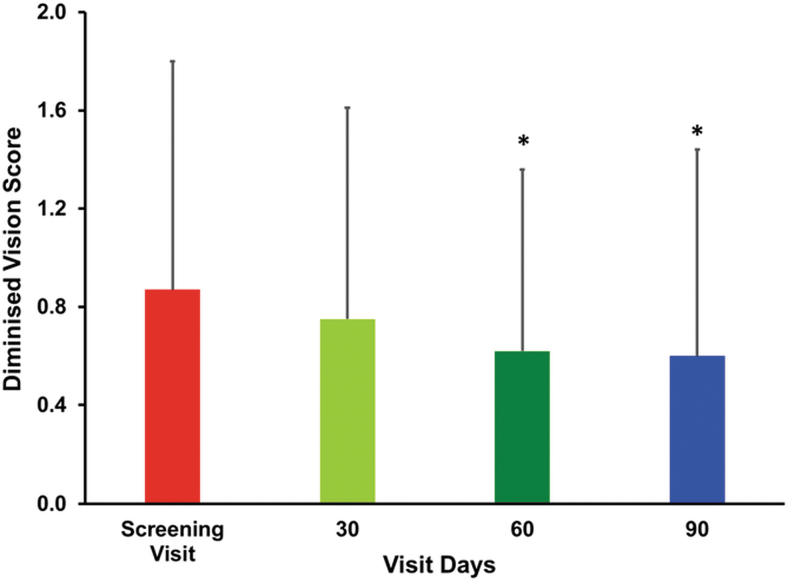
The bar graph shows the mean change in diminished vision scores. The diminished vision was assessed according to the grading of subjects' scores on screening visits (baseline) and after treatment on days 30, 60, and 90 to assess the vision. Values are presented as mean ± SD. Pairwise “*t*” test was used for statistical comparison. The change in diminished vision scores of subjects after treatment on 60 and 90 days was found to be statistically significant, *P < .05. SD, standard deviation.

**FIG. 3.**
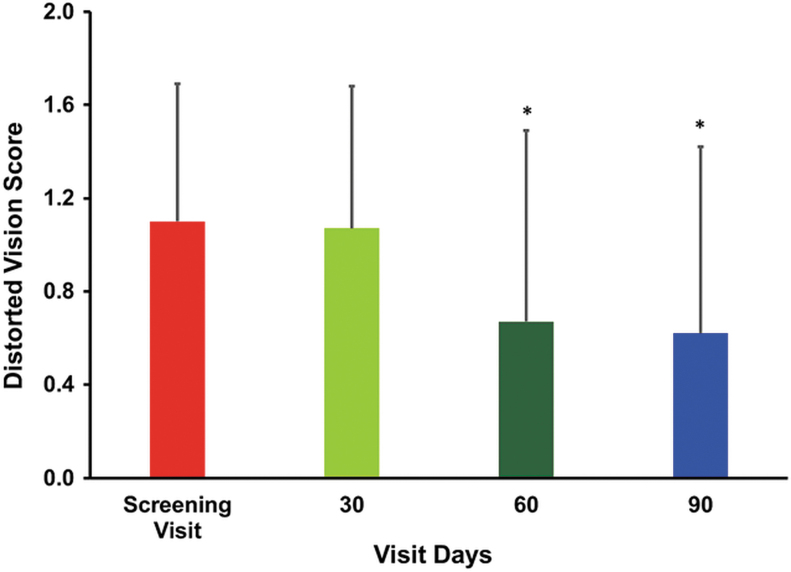
The bar graph shows the mean change in distortion vision impairment. Distortion vision was assessed on screening visits (baseline) and after treatment on days 30, 60, and 90. Values are presented as mean ± SD. Pairwise “*t*” test was used for statistical comparison. The change in distorted vision scores of subjects after treatment on 60 and 90 days was found to be statistically significant, *P < .05.

The difficulty in day vision scores (mean ± SD) showed progressive improvement, 1.07 ± 0.26, 1.07 ± 0.26, and 1.05 ± 0.45 after treatment of 30, 60, and 90 days, respectively, compared with the baseline (1.17 ± 0.38). However, the change in the difficulty in day vision score (0.12 ± 0.68) observed at the end of the study was found to be not statistically significant (Table 3). In the case of diminished vision, the mean scores decreased gradually from baseline values of 0.87 ± 0.93 to 0.75 ± 0.86, 0.62 ± 0.74, and 0.6 ± 0.84 after treatment of 30, 60, and 90 days, respectively. The change in diminished vision scores 0.15 ± 0.54 and 0.27 ± 0.64 observed on day 60 and 90 from the baseline was found to be statistically significant. Thus, treatment with the study supplement significantly improved the diminished vision scores, (*P* < .05) (Fig. 2). Similarly, the result signifies a progressive improvement in the distorted vision after treatment over a period of 60 and 90 days. The mean distorted vision scores decreased steadily from the baseline value of 1.1 ± 0.5 to 1.07 ± 0.61, 0.67 ± 0.82, and 0.62 ± 0.8 on days 30, 60, and 90, respectively. The changes observed after treatment in the distorted vision scores on days 60 and 90 were found to be statistically significant (*P* < .05) (Fig. 3**)**. There was an improvement in the perception of black spots. The mean ± SD score decreased gradually from the baseline value of 0.62 ± 0.77 to 0.52 ± 0.64, 0.47 ± 0.55, and 0.45 ± 0.67 on days 30, 60, and 90, respectively. However, the change in the perception of black spots score was found to be not statistically significant. Similar trends were also observed in the case of dark adaptation scores, as well as in the scores of perceptions of flashes of light and objects as yellowish (Table 3). The findings of the study showed no worsening in the parameters just mentioned related to early-stage dry-type AMD.

**Table 3.** Improvement in Subjective Symptom Scores of Subjects After Treatment

Subjective symptoms	Screening (baseline)	Day 30	Day 60	Day 90
	(Mean ± SD)	(Mean ± SD)	(Mean ± SD)	(Mean ± SD)
Difficulty in day vision	1.17 ± 0.38	1.07 ± 0.26	1.07 ± 0.26	1.05 ± 0.45
Change from screening		0.1 ± 0.37	0.1 ± 0.44	0.12 ± 0.68
Perception of black spots	0.62 ± 0.77	0.52 ± 0.64	0.47 ± 0.55	0.45 ± 0.67
Change from screening		0.1 ± 0.37	0.15 ± 0.42	0.17 ± 0.67
Dark adaptation	0.72 ± 0.87	0.67 ± 0.82	0.62 ± 0.77	0.6 ± 0.81
Change from screening		0.05 ± 0.31	0.10 ± 0.44	0.12 ± 0.64

Data presented as mean ± SD. Pairwise “*t*” test was used for statistical comparison. All subjective symptom scores showed progressive improvement after treatment, but they are found to be not statistically significant.

### Secondary efficacy outcomes

The distant vision was assessed by using the Snellen chart at screening and on days 30, 60 and 90. A maximum number of 25% of the subjects had visual acuity of 6/18 and 22.5% had visual acuity of 6/9, 6/24 for far-left eye vision (uncorrected) and 57.5% had visual acuity of 6/9 (corrected) at baseline. Uncorrected and corrected vision of far-right eye at baseline was found to be 6/18 (27.5%) and 6/9 (57.5%), respectively. After treatment, the maximum uncorrected and corrected vision among patients with far-left eye was found to be 6/18 (25%) and 6/9 (55%), respectively, on day 90. The maximum uncorrected and corrected vision among patients with far-right eye was 6/18 (27.8%) and 6/9 (57.5%), respectively, on day 90 (Supplementary Table S2 and S3). The findings of this study revealed no worsening of eye condition after 90 days of treatment.

A pinhole occluder is being used to test visual acuity. A maximum number of patients, that is, 55% had pinhole vision of 6/9, far-right eye vision (uncorrected) and 55% had pinhole vision of 6/9 (corrected) at baseline. Uncorrected and corrected vision of far-left eye at baseline was found to be 6/9 (55%) and 6/9 (57.5%), respectively. After treatment, the maximum uncorrected and corrected pinhole vision among patients with far-right eye was found to be 6/9 (57.5%) and 6/12 (57.5%), respectively, on day 90. The maximum uncorrected and corrected vision among patients with far-left eye was found to be 6/9 (57.5%) and 6/12 (60%), respectively, on day 90 (Supplementary Table S4). The findings reveal that the study supplement seems to preserve the eye condition without further deterioration.

**FIG. 4.**
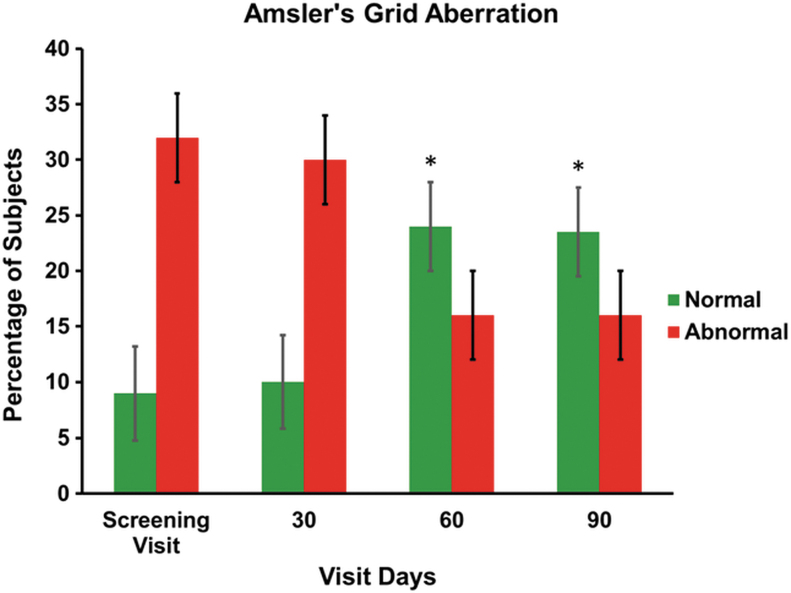
The bar graph shows the percentage change of Amsler's grid aberration in subjects on screening visits (baseline) and after treatment on days 30, 60, and 90. Values are presented as mean ± SD. Pairwise “*t*” test was used for statistical comparison. The change after treatment on 60 and 90 days was found to be statistically significant, *P < .05.

Dilated fundus examination showed the presence of drusen at the beginning and at the end of the study, revealing no change after treatment. There was also no major change in OCT observation at the end of the study on day 90. Amsler's grid test is one of the most common techniques performed in patients to monitor the health of the macula. The Amsler's grid showed a progressive improvement in the vision (Fig. 4). At the time of screening, 77.5% of subjects had abnormal Amsler's grid aberration scores and at the end of the treatment (day 90), the score was significantly reduced in 60% of the subjects. The changes in the scores after treatment on days 60 and 90 from the baseline were found to be statistically significant (*P* < .05). The Humphrey visual field score after treatment was found to be normal throughout the study period.

### Safety outcomes

All the 40 subjects completed the study. There were no AEs recorded in the study population. All mean vital signs values were within the reference ranges. No significant changes in the vital signs or the laboratory parameters were observed among the study population (Supplementary Table S5).

## DISCUSSION

AMD leads to a loss of central vision due to degeneration of photoreceptors in the macula. Even though a variety of supplements are being used for the treatment of AMD, several limitations have been reported on the safety and efficacy of the existing formulations. The AREDS study suggests that the dietary intake of lutein and zeaxanthin reduced the risk of AMD. Although the AREDS supplements were beneficial, a few adverse effects were reported.^8^ Results of the subsequent AREDS2 trial were conducted by using lutein+zeaxanthin and omega-3 fatty acids in the AREDS formulation while replacing *β*-carotene; however, it did not reduce the progression to advanced AMD.^9^ Similarly, in the LAST with lutein and mixed antioxidant formulation, minor side effects were observed with patients who received the mixed formulation of lutein/antioxidants/vitamins/minerals.^11^

This study demonstrated that Macumax^®^ supplementation could prevent the disease from worsening in patients with early-stage dry-type AMD without causing AEs. Lutein and its isomer zeaxanthin are the major dietary xanthophyll carotenoids that accumulate in the macular region of the human retina and that are responsible for sharp, central vision and enhancing visual acuity, as well as for protecting the macula from damaging blue light and harmful reactive oxygen species (ROS).^16^

Traditionally, lutein is characterized by its blue light filtering and antioxidant properties.^17^ Lutein supplementation has been shown to decrease the circulating complementary proinflammatory components C5a, C3d, and Complement Factor D (CFD) of the intrinsic immune system^18^ and a soluble complement membrane attack complex (sC5b-9), a mediator of inflammation associated with the development of AMD.^18^ Further, recent studies using animal models show that lutein exerts neuroprotective effects against retinal neural damage caused by inflammation and protects the photoreceptor cell function in the inflamed retinas.^19^

*In vivo* experimental studies carried out by Thomson, Toyoda, Langner, Delori, Garnett, Craft, Nichols, Cheng, Dorey^20^ showed that zeaxanthin supplementation prevented light-induced photoreceptor cell death in quail by enhancing the retinal zeaxanthin in a dose-dependent manner. Dietary zeaxanthin also has been shown to prevent oxidative stress in RPE and possibly reduced the progression of AMD.^21^ Earlier studies support the selective uptake of lutein and zeaxanthin in the retina, suggesting their important role in the maintenance of ocular health.^22^ Higher dietary lutein and zeaxanthin intake reduces the incidence of AMD, whereas their low levels are associated with AMD.^23^ Further, the administration of lutein and zeaxanthin has been shown to protect retinal cell damage in diabetic retinopathy.^24^ Supplementation with lutein and/or zeaxanthin has been shown to enhance retinal sensitivity in patients with early AMD.^25^ The levels of lutein and zeaxanthin in the macula of the primate retina concentrations are 500-times greater than other body tissues and are presumed to be neuroprotective as blue-light filters and antioxidants.^26^

Piperine from black pepper fruits (*Piper nigrum*) has been used as a bioavailability enhancer for a number of phytochemicals.^27^ Piperine blended with lutein and zeaxanthin in an optimized ratio has been shown to be beneficial to eye health.^28^

Bilberries (*Vaccinium myrtillus*) are a rich source of anthocyanins, recognized for their importance in eye health. Earlier studies used commercially available bilberry extract for the improvement of night vision in normal individuals.^29^ Recent studies show that bilberry extract ameliorates ocular symptoms and has been used in clinical trials for eye fatigue.^30^ Studies using *in vitro* experiments show that it reduces ROS^31^ and suppresses the pathogenesis of innate retinal inflammation.^32^ In addition, bilberry extract has been shown to reduce photo-induced apoptosis and visual dysfunction partly by decreasing ROS and endoplasmic reticulum stress in the retina in a murine model,^33^ suggesting that bilberry extract could be potentially useful in therapeutic approach for preventing retinal photo-damage.

Zinc plays an integral role in maintaining normal ocular function.^34^ Zinc monomethionine has been suggested as a novel dietary supplement since it has superior bioavailability than other forms of zinc with antioxidant and immune-enhancing properties.^35^ Piperine acts as a bioavailability enhancer for minerals such as Zinc.^28^

A recent longitudinal, open-label clinical study^36^ evaluated the effect of Saffron in early AMD patients over a longer duration and showed that Saffron supplementation improved macular function. In a subsequent study, the same group^37^ showed that the long-term efficacy of Saffron in improving macular function has direct implications for the clinical management of early AMD.

Regarding clinical efficacy, Macumax^®^ treatment showed improvements in difficulty in day vision scores, perception of black spots, and improvement in dark adaptation compared with baseline. There were significant improvements in the diminished and distorted vision scores, respectively (*P* < .05). Similarly, improvements were also observed with secondary efficacy endpoints, namely in distant vision measures with Snellen visual acuity, pinhole vision, fundus changes, and in OCT. There was a significant change in Amsler's Grid test, which confirms a gradual improvement in the pathological condition. The percentage improvement in Amsler's Grid test was found to be 25% on day 30 and 60% on days 60 and 90 after the treatment. The change is found to be significant as confirmed from a distorted vision, which was assessed with the use of Amsler's grid (*P* < .05). Thus, the results of this trial confirm that the use of the study supplement preserves the eye condition without further deterioration and it can be concluded that with long usage it will prevent or delay the vision loss that has been observed if AMD is not treated. Out of 40 subjects, none of the subjects reported any AEs during the entire study period.

There are some limitations in the current study, namely the use of a small group of subjects, shorter duration of the study period, and lack of a control group. Although the results of this open-label study are encouraging, future clinical trials need to be designed, including a control group, and conducted for a longer period, at least over 6 months to accurately determine the effectiveness of the study supplement. Another limitation is the lack of information on the influence of individual variations of the dietary habits and contents (especially those rich in antioxidants and micronutrients) that could impact the results.

In conclusion, the findings provide clinical evidence that a 90-day oral supplementation of Macumax^®^ was safe and helpful in preventing early-stage dry-type AMD without further progression of the disease.

## ETHICAL APPROVAL

The study was performed in accordance with good clinical practices as required by the International Conference on Harmonization, local regulatory requirement, and standard operating procedures for clinical investigation and in compliance with the Declaration of Helsinki. The trial was conducted as per the protocol approved by the Institutional Ethics Committee without any deviation. The study was registered as a clinical trial (CTRI/2016/02/006676) at a public Clinical Trial Registry in India (www.ctri.nic.in).^14^ Written informed consent was obtained from all the participants of this trial.

## Supplementary Material

Supplemental data

Supplemental data

Supplemental data

Supplemental data

Supplemental data
