# Supplementary material for: An Open-Label Pilot Study on Macumax Supplementation for Dry-Type Age-Related Macular Degeneration
Source: J Med Food. 2021 May 17;24(5):551–7. doi: 10.1089/jmf.2020.0097 (PMC8140349; doi:10.1089/jmf.2020.0097)
Supplement: Supplemental data [file Supp_Table1.docx]

**Majeed et al.**

**Supplementary Table 1.** Summary of inclusion and exclusion criteria.

**Inclusion criteria**

1. Patients of either sex, >50 years of age.
2. (b) Patients having difficulty in distance vision but no interference with routine work (Score 1) that are signs and symptoms of early (primary) form of dry AMD
3. willing to come for regular follow-up visits and
4. able to give written informed consent.

**Exclusion criteria:**

Subjects who had any of the following conditions at screening were excluded from enrolment:

1. Subjects having normal vision (no difficulty in day vision, Score 0)
2. Subjects having occasionally interference with day to day working (Score 2) and cannot do any work in bright daylight that are more advanced stage dry-type AMD.
3. Subjects with both neovascular (wet AMD) and GA that are the advanced form of AMD
4. Patients with a known history of hypersensitivity to herbal extracts or dietary supplements
5. Patients with glaucoma, high myopia, mature cataract, pan-retinal degenerations, etc.
6. Patients with hypercholesterolemia, renal disorder, liver disease, and other debilitating illnesses.
7. Women with the following conditions: pregnant, lactating, childbearing potential and found positive for urine pregnancy testing.
8. Subjects with on-going treatment with herbals or allopathic ocular drugs.
9. Subjects with a history of having received any investigational drug or enrolled in currently ongoing clinical studies or participated in any other clinical trial that was completed in the previous month.
10. Any illness that in the investigator’s opinion does not warrant the inclusion of the patient in the study.
