# Supplementary material for: An Open-Label Pilot Study on Macumax Supplementation for Dry-Type Age-Related Macular Degeneration
Source: J Med Food. 2021 May 17;24(5):551–7. doi: 10.1089/jmf.2020.0097 (PMC8140349; doi:10.1089/jmf.2020.0097)
Supplement: Supplemental data [file Supp_Table2.docx]

**Majeed et al.**

**Supplementary Table 2.** Visual acuity of far vision (left and right eye) of subjects recorded at screening and after treatment

| **Visual Acuity** | **Far Vision for Left eye** | | **Far Vision for Right eye** | |
| --- | --- | --- | --- | --- |
|  | **Uncorrected** | **Corrected** | **Uncorrected** | **Corrected** |
| **Screening visit** | | | | |
| 6/12 | 7 (17.5%) | 11 (27.5%) | 8 (20%) | 13 (32.5%) |
| 6/9 | 9 (22.5%) | 23 (57.5%) | 8 (20%) | 23 (57.5%) |
| 6/18 | 10 (25%) | 3 (7.5%) | 11 (27.5%) | 2 (5%) |
| 6/24 | 9 (22.5%) | 1 (2.5%) | 7 (17.5%) | 7 (17.5%) |
| 6/36 | 1 (2.5%) | - | 2 (5%) | - |
| 6/60 | 4 (10%) | - | 4 (10%) | - |
| 6/6 | - | 2 (5%) | - | - |
| **Baseline** | | | | |
| 6/12 | 7 (17.5%) | 11 (27.5%) | 8 (20%) | 13 (32.5%) |
| 6/9 | 9 (22.5%) | 23 (57.5%) | 8 (20%) | 23 (57.5%) |
| 6/18 | 10 (25%) | 3 (7.5%) | 11 (27.5%) | 2 (5%) |
| 6/24 | 9 (22.5%) | 1 (2.5%) | 7 (17.5%) | 7 (17.5%) |
| 6/36 | 1 (2.5%) | - | 2 (5%) | - |
| 6/60 | 4 (10%) | - | 4 (10%) | - |
| 6/6 | - | 2 (5%) | - | - |
| **Day 30** | | | | |
| 6/12 | 7 (17.5%) | 11 (27.5%) | 8 (20%) | 13 (32.5%) |
| 6/9 | 9 (22.5%) | 23 (57.5%) | 8 (20%) | 23 (57.5%) |
| 6/18 | 10 (25%) | 3 (7.5%) | 11 (27.5%) | 2 (5%) |
| 6/24 | 9 (22.5%) | 1 (2.5%) | 7 (17.5%) | 2 (5%) |
| 6/36 | 1 (2.5%) | - | 2 (5%) | - |
| 6/60 | 4 (10%) | - | 4 (10%) | - |
| 6/6 | - | 2 (5%) | - | - |
| **Day 60** | | | | |
| 6/12 | 7 (17.5%) | 11 (27.5%) | 8 (20%) | 13 (32.5%) |
| 6/9 | 9 (22.5%) | 23 (57.5%) | 8 (20%) | 23 (57.5%) |
| 6/18 | 10 (25%) | 3 (7.5%) | 11 (27.5%) | 2 (5%) |
| 6/24 | 9 (22.5%) | 1 (2.5%) | 7 (17.5%) | 2 (5%) |
| 6/36 | 1 (2.5%) | - | 2 (5%) | - |
| 6/60 | 4 (10%) | - | 4 (10%) | - |
| 6/6 | - | 2 (5%) | - | - |
| **Day 90** | | | | |
| 6/12 | 8 (20%) | 12 (30%) | 8 (20%) | 13 (32.5%) |
| 6/9 | 8 (20%) | 22 (55%) | 8 (20%) | 23 (57.5%) |
| 6/18 | 10 (25%) | 3 (7.5%) | 11 (27.5%) | 2 (5%) |
| 6/24 | 9 (22.5%) | 1 (2.5%) | 7 (17.5%) | 2 (5%) |
| 6/36 | 1 (2.5%) | - | 2 (5%) | - |
| 6/60 | 4 (10%) | - | 4 (10%) | - |
| 6/6 | - | 2 (5%) | - | - |
