# Supplementary material for: An Open-Label Pilot Study on Macumax Supplementation for Dry-Type Age-Related Macular Degeneration
Source: J Med Food. 2021 May 17;24(5):551–7. doi: 10.1089/jmf.2020.0097 (PMC8140349; doi:10.1089/jmf.2020.0097)
Supplement: Supplemental data [file Supp_Table3.docx]

**Majeed et al.**

**Supplementary Table 3.** Visual acuity for near vision (left and right eye) of subjects recorded at screening and after treatment

| **Visual Acuity** | **Near Left Eye** | | **Near Right Eye** | |
| --- | --- | --- | --- | --- |
|  | **Uncorrected** | **Corrected** | **Uncorrected** | **Corrected** |
| **Screening visit** | | | | |
| N24 | 4 (10%) | - | 5 (12.5%) | - |
| N36 | 18 (45%) | 2 (5%) | 17 (42.5%) | 2 (5%) |
| N18 | 14 (35%) | - | 14 (35%) | - |
| N8 | 4 (10%) | 9 (22.5%) | 4 (10%) | 11 (27.5%) |
| N10 | - | 2 (5%) | - | - |
| N6 | - | 27 (67.5%) | - | 27 (67.5%) |
| **Baseline visit** | | | | |
| N24 | 4 (10%) | - | 5 (12.5%) | - |
| N36 | 18 (45%) | 2 (5%) | 17 (42.5%) | 1 (2.5%) |
| N18 | 14 (35%) | - | 14 (35%) | - |
| N8 | 4 (10%) | 9 (22.5%) | 4 (10%) | 11 (27.5%) |
| N10 | - | 2 (5%) | - | - |
| N6 |  | 27 (67.5%) | - | 28 (70%) |
| **Day 30** | | | | |
| N24 | 4 (10%) | - | 5 (12.5%) | - |
| N36 | 18 (45%) | 2 (5%) | 17 (42.5%) | 2 (5%) |
| N18 | 14 (35%) | - | 14 (35%) | - |
| N8 | 4 (10%) | 9 (22.5%) | 4 (10%) | 10 (25%) |
| N10 | - | 2 (5%) | - | - |
| N6 | - | 27 (67.5%) | - | 28 (70%) |
| **Day 60** | | | | |
| N24 | 4 (10%) | - | 5 (12.5%) | - |
| N36 | 18 (45%) | 2 (5%) | 17 (42.5%) | 2 (5%) |
| N18 | 14 (35%) | - | 14 (35%) | - |
| N8 | 4 (10%) | 9 (22.5%) | 4 (10%) | 11 (27.5%) |
| N10 | - | 2 (5%) | - | - |
| N6 | - | 27 (67.5%) | - | 27 (67.5%) |
| **Day 90** | | | | |
| N24 | 4 (10%) | - | 5 (12.5%) | - |
| N36 | 18 (45%) | 1 (2.5%) | 17 (42.5%) | 1 (2.5%) |
| N18 | 14 (35%) | - | 14 (35%) | - |
| N8 | 4 (10%) | 9 (22.5%) | 4 (10%) | 10 (25%) |
| N10 | - | 2 (5%) | - | - |
| N6 | - | 28 (70%) | - | 28 (70%) |
| N12 | - | - | - | 1 (2.5%) |
