# Supplementary material for: An Open-Label Pilot Study on Macumax Supplementation for Dry-Type Age-Related Macular Degeneration
Source: J Med Food. 2021 May 17;24(5):551–7. doi: 10.1089/jmf.2020.0097 (PMC8140349; doi:10.1089/jmf.2020.0097)
Supplement: Supplemental data [file Supp_Table4.docx]

**Majeed et al.**

**Supplementary Table 4**. Pinhole vision of subjects recorded at screening and after treatment

| Pin hole Vision | OD | | PH | | OS | | PH | |
| --- | --- | --- | --- | --- | --- | --- | --- | --- |
|  | Corrected | Uncorrected | Corrected | Uncorrected | Corrected | Uncorrected | Corrected | Uncorrected |
| **Screening** | | | | | | | | |
| 6/9 | 9 (22.5%) | 8(20%) | 22(55%) | 22(55%) | 11(27.5%) | 9(22.5%) | 23(57.5%) | 22(55%) |
| 6/12 | 13(32.5%) | 9(22.5%) | 13(32.5%) | 12(30%) | 11(27.5%) | 8(20%) | 11(27.5%) | 12(30%) |
| 6/18 | 6(15) | 10(25%) | 2(5%) | 3(7.5%) | 6(15%) | 9(22.5%) | 3(7.5%) | 3(7.5%) |
| 6/24 | 7(17.5%) | 7(17.5%) | 2(5%) | 2(5%) | 7(17.5%) | 9(22.5%) | 1(2.5%) | 1(2.5%) |
| 6/36 | 1(2.5%) | 2(5%) | - | - | 1(2.5%) | 1(2.5%) | - | - |
| 6/60 | 4(10%) | 4(10%) | - | - | 4(10%) | 4(10%) | - | - |
| 6/6 | - | - | 1(2.5%) | 1(2.5%) | - | - | 2(5%) | 2(5%) |
| **Baseline** | | | | | | | | |
| 6/9 | 9(22.5%) | 8(20%) | 23(57.5%) | 23(57.5%) | 11(27.5%) | 9(22.5%) | 24(60%) | 23(57.5%) |
| 6/12 | 14(35%) | 8(20%) | 13(32.5%) | 12(30%) | 11(27.5%) | 6(15%) | 11(27.5%) | 12(30%) |
| 6/18 | 5(12.5%) | 12(30%) | 2(5%) | 3(7.5%) | 6(15%) | 12(30%) | 3(7.5%) | 3(7.5%) |
| 6/24 | 7(17.5%) | 6(15%) | 2(5%) | 2(5%) | 7(17.5%) | 8(20%) | 1(2.5%) | 1(2.5%) |
| 6/36 | 1(2.5%) | 2(5%) | - | - | 1(2.5%) | 1(2.5%) | - | - |
| 6/60 | 4(10%) | 4(10%) | - | - | 4(10%) | 4(10%) | - | - |
| 6/6 | - | - | - | - | - | - | 1(2.5%) | 1(2.5%) |
| **Day 30** | | | | | | | | |
| 6/9 | 9(22.5%) | 8(20%) | 23(57.5%) | 23(57.5%) | 11(27.5%) | 9(22.5%) | 24(60%) | 23(57.5%) |
| 6/12 | 13(32.5%) | 9(22.5%) | 13(32.5%) | 12(30%) | 11(27.5%) | 8(20%) | 11(27.5%) | 12(30%) |
| 6/18 | 6(15%) | 10(25%) | 2(5%) | 3(7.5%) | 6(15%) | 9(22.5%) | 4(10%) | 4(10%) |
| 6/24 | 7(17.5%) | 7(17.5%) | 2(5%) | 2(5%) | 7(17.5%) | 9(22.5%) | 1(2.5%) | 1(2.5%) |
| 6/36 | 1(2.5%) | 2(5%) | - | - | 1(2.5%) | 1(2.5%) | - | - |
| 6/60 | 4(10%) | 4(10%) | - | - | 4(10%) | 4(10%) | - | - |
| 6/6 | - | - | - | - | - | - | - | - |
| **Day 60** | | | | | | | | |
| 6/9 | 9 (22.5%) | 8(20%) | 23(57.5%) | 23(57.5%) | 9(22.5%) | 9(22.5%) | 24(60%) | 23(57.5%) |
| 6/12 | 13(32.5%) | 9(22.5%) | 13(32.5%) | 12(30%) | 8(20%) | 8(20%) | 11(27.5%) | 12(30%) |
| 6/18 | 6(15%) | 10(22.5%) | 2(5%) | 3(7.5%) | 5(12.5%) | 9(22.5%) | 3(7.5%) | 3(7.5%) |
| 6/24 | 7(17.5%) | 7(17.5%) | 2(5%) | 2(5%) | 16(40%) | 9(22.5%) | 1(2.5%) | 1(2.5%) |
| 6/36 | 1(2.5%) | 2(5%) | - | - | 1(2.5%) | 1(2.5%) | - | - |
| 6/60 | 4(10%) | 4(10%) | - | - | 1(2.5%) | 4(10%) | - | - |
| 6/6 | - | - | - | - | - | - | 1(2.5%) | 1(2.5%) |
| **Day 90** | | | | | | | | |
| 6/9 | 9 (22.5%) | 8(20%) | 23(57.5%) | 23(57.5%) | 11(27.5%) | 9(22.5%) | 24(60%) | 23(57.5%) |
| 6/12 | 13(32.5%) | 10(25%) | 13(32.5%) | 12(30%) | 11(27.5%) | 9(22.5%) | 11(27.5%) | 12(30%) |
| 6/18 | 6(15%) | 7(17.5%) | 2(5%) | 3(7.5%) | 6(15%) | 8(20%) | 3(7.5%) | 3(7.5%) |
| 6/24 | 7(17.5%) | 7(17.5%) | 2(5%) | 2(5%) | 7(17.5%) | 9(22.5%) | 1(2.5%) | 1(2.5%) |
| 6/36 | 1(2.5%) | 2(5%) | - | - | 1(2.5%) | 1(2.5%) | - | - |
| 6/60 | 4(10%) | 4(10%) | - | - | 4(10%) | 4(10%) | - | - |
| 6/6 | - | - | - | - | - | - | 1(2.5%) | 1(2.5%) |
