# Supplementary material for: An Open-Label Pilot Study on Macumax Supplementation for Dry-Type Age-Related Macular Degeneration
Source: J Med Food. 2021 May 17;24(5):551–7. doi: 10.1089/jmf.2020.0097 (PMC8140349; doi:10.1089/jmf.2020.0097)
Supplement: Supplemental data [file Supp_Table5.docx]

**Majeed et al.**

**Supplementary Table 5.** Change in vital signs of subjects recorded from Baseline to the end of the study

| **Parameter** | **Statistics**  **(N)** | **Screening**  **(40)** | **Baseline**  **(40)** | **Day 30**  **(40)** | **Day 60**  **(40)** | **Day 90**  **(40)** | **Change from Screening** |
| --- | --- | --- | --- | --- | --- | --- | --- |
| **Pulse Rate**  **(beats/min)** | Mean (S.D) | 81.4 (6.4) | 80.825 (6.7) | 79.8 (5.1) | 82.275 (5.4) | 82.8 (7.0) | 1.4 |
|  | Median | 80.5 | 80 | 80 | 80 | 80 |  |
|  | Min; Max | 72, 98 | 72, 98 | 72, 98 | 75, 98 | 72, 98 |  |
| **Respiratory rate (breaths/min) per minute)** | Mean (S.D) | 19.5 (1.9) | 19.3 (1.6) | 19.425 (1.5) | 19.4 (1.7) | 19.175 (1.4) | -0.325 |
|  | Median | 20 | 19 | 20 | 19 | 19 |  |
|  | Min; Max | 14, 22 | 15, 22 | 16, 22 | 16, 22 | 16, 22 |  |
| **Heart rate (beats/min)** | Mean (S.D) | 81.075 (6.4) | 80.575 (6.7) | 79.85 (5.2) | 82.35 (5.4) | 82.75 (7.1) | 1.68 |
|  | Median | 80 | 80 | 80 | 81 | 80 |  |
|  | Min; Max | 74, 98 | 72, 98 | 72, 98 | 76, 98 | 72, 98 |  |
| **Systolic Blood pressure** | Mean (S.D) | 124.975 (9.1) | 125.45 (7.4) | 124.125 (6.0) | 122.425 (6.1) | 123 (6.5) | -1.97 |
|  | Median | 123 | 123 | 122 | 121 | 122 |  |
|  | Min; Max | 110,145 | 112, 143 | 110, 140 | 110, 132 | 106, 132 |  |
| **Diastolic Blood pressure** | Mean (S.D) | 80.55 (6.0) | 81.05 (5.5) | 78.575 (4.0) | 79.4 (3.7) | 78.975 (4.4) | -1.58 |
|  | Median | 80 | 80 | 80 | 80 | 80 |  |
|  | Min; Max | 70, 94 | 72, 92 | 67, 90 | 70, 90 | 68, 90 |  |

Data presented as mean±SD.

Pairwise ‘t’ test was used for statistical comparison.

The change from screening and after treatment in the clinical parameters was found to be not statistically significant.
